# Role of Known Transient Receptor Potential Vanilloid Channels in Modulating Cardiac Mechanobiology

**DOI:** 10.3389/fphys.2021.734113

**Published:** 2021-10-15

**Authors:** Michael Miller, Sheryl E. Koch, Adam Veteto, Timothy Domeier, Jack Rubinstein

**Affiliations:** ^1^Department of Medical Pharmacology and Physiology, University of Missouri School of Medicine, Columbia, KY, United States; ^2^Department of Internal Medicine, Division of Cardiovascular Health and Disease, College of Medicine, University of Cincinnati Medical Center, Cincinnati, OH, United States; ^3^IonOptix, LLC, Westwood, MA, United States; ^4^Division of Cardiovascular Medicine, Cincinnati Veterans Affairs Medical Center, Cincinnati, OH, United States

**Keywords:** TRPV channels, hypertrophy, cardiomyopathies, fibrosis, cardiac fibrosis

## Abstract

The transient receptor potential (TRP) channels have been described in almost every mammalian cell type. Several members of the Vanilloid (TRPV) subtype have been found to play important roles in modulating cardiac structure and function through Ca^2+^ handling in response to systemic and local mechanobiological cues. In this review, we will consider the most studied TRPV channels in the cardiovascular field; transient receptor potential vanilloid 1 as a modulator of cardiac hypertrophy; transient receptor potential vanilloid 2 as a structural and functional protein; transient receptor potential vanilloid 3 in the development of hypertrophy and myocardial fibrosis; and transient receptor potential vanilloid 4 in its roles modulating the fibrotic and functional responses of the heart to pressure overload. Lastly, we will also review the potential overlapping roles of these channels with other TRP proteins as well as the advances in translational and clinical arenas associated with TRPV channels.

## Introduction

Since their initial discovery in the late 1990s, the transient receptor potential (TRP) family of channels has been described in almost every mammalian cell type (Nilius and Szallasi, 2014). Of these, several members of the Vanilloid (TRPV) subtype have been found to play important roles in cardiac physiology under both physiologic and pathologic conditions in their response to systemic and local mechanobiological cues.

The TRPV channels were initially described in the neurology field as transmitters of pain and stretch stimuli (Montell, 2005; Cortright et al., 2007; Venkatachalam and Montell, 2007). Specifically, as mechanosensors they were found to open in response to sufficient intracellular or extracellular forces initiating rapid cellular responses. Furthermore, these channels have generally been found to be upregulated under persistent stress conditions, thus also modulating the cellular response following subacute and chronic stress.

**Figure 1:**
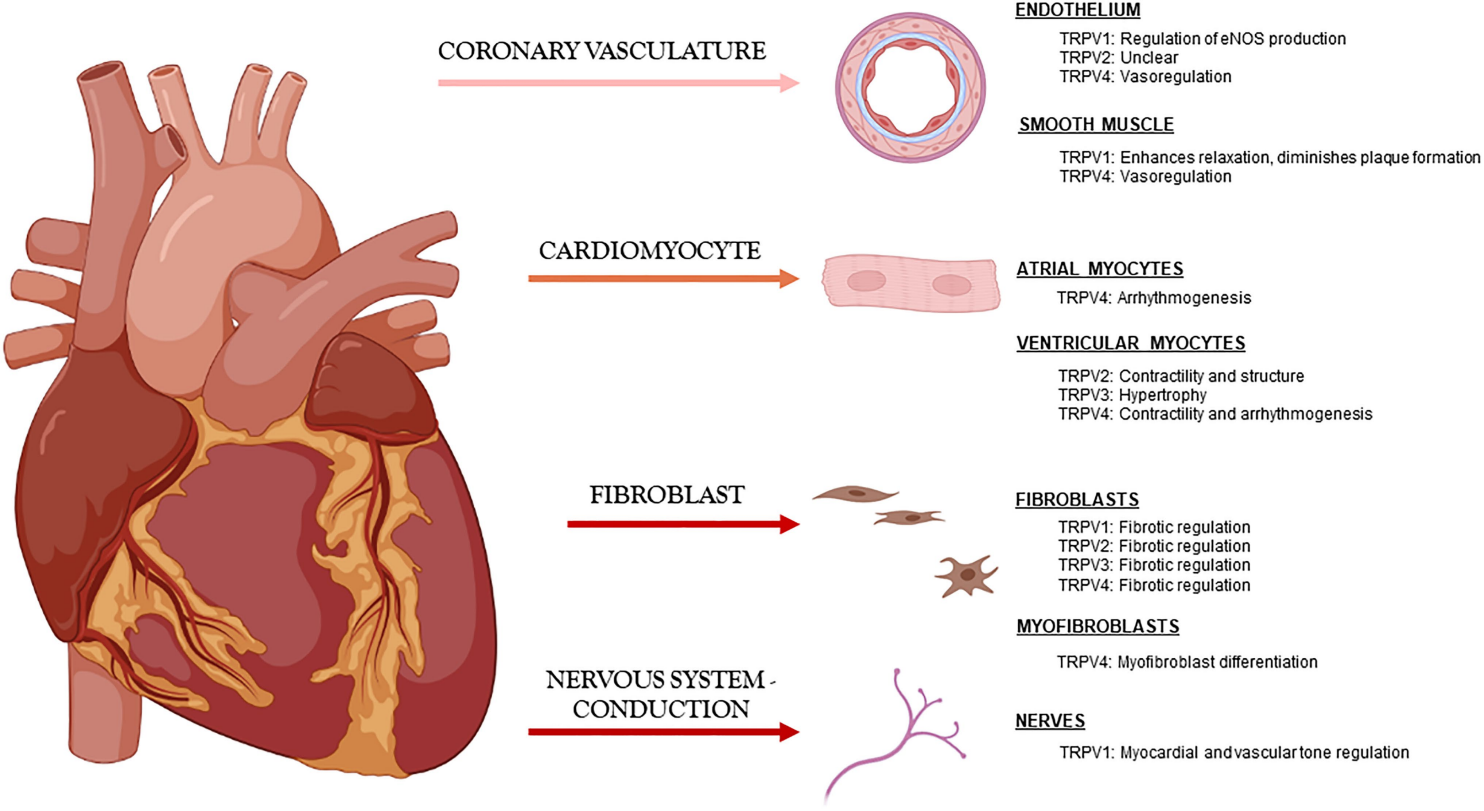
Known roles of TRPV channels in cardiac tissue under physiologic conditions.

The most studied TRPV channels in the cardiovascular field are 1–4, and although mostly studied independently by laboratories around the world, it has become clearer that they play similar and occasionally overlapping roles in maintaining cardiac function through modulation of Ca^2+^ handling and also in maintaining cardiac structure directly through myocyte-myocyte interactions and indirectly through myocyte-fibroblast communication (Figure 1).

**Figure 2:**
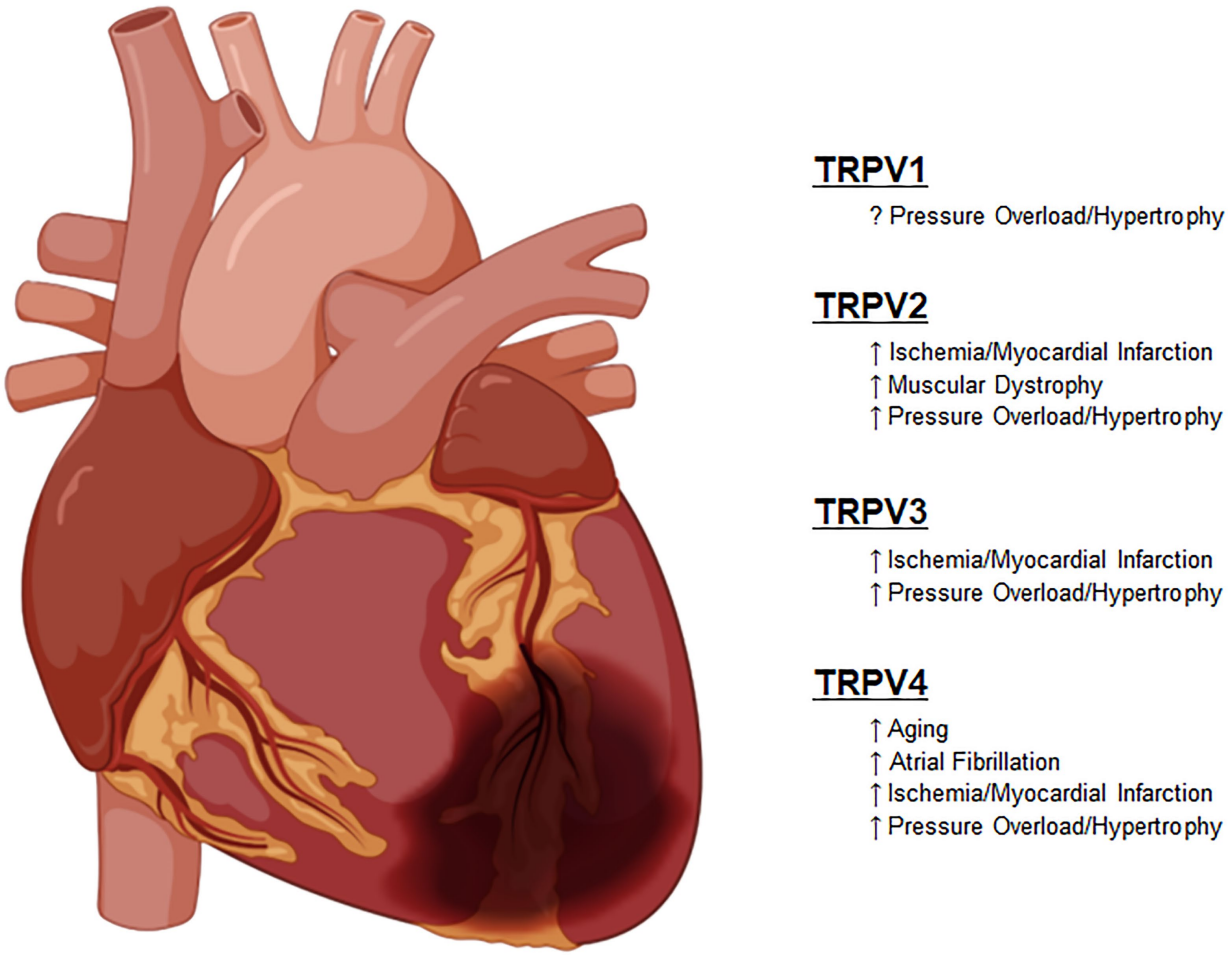
Known roles of TRPV channel responses to pathologic stimuli.

These important functions have not escaped the attention of translational and clinical scientists that have attempted to harness these newly discovered functions into clinically relevant therapies. As described in each section in detail, these studies have sought to improve outcomes in diseases, such as heart failure with both reduced and preserved ejection fraction (HFrEF and HFpEF), various forms of cardiomyopathies and in some cases for prevention of pre-clinical compensatory mechanisms, such as left ventricular hypertrophy (Figure 2).

## Transient Receptor Potential Vanilloid 1

The transient receptor potential cation channel subfamily Vanilloid member 1 (TRPV1) was the first of the Vanilloid subfamily to be identified in Caterina et al. (1997). It was originally described as the nociceptive capsaicin receptor as it exhibits activation by capsaicin, an active chemical irritant found in chili peppers. TRPV1 is a non-selective ion channel with significant permeability to Ca^2+^ and Na^+^ and less selectivity for H^+^ and Mg^2+^. Structurally, TRPV1 consists of four identical subunits each with six transmembrane domains and a loop helix between the fifth and sixth domain (Randhawa and Jaggi, 2017; Szabados et al., 2020). This channel has a dual gating mechanism that remains closed in absence of activators. The first gate is an ion selectivity filter on the external region of the pore and the second is the S6 helices along the cytosolic side of the pore (Jara-Oseguera et al., 2019).

In addition to capsaicin, TRPV1 is activated by numerous other stimuli, such as compounds produced from hypoxic conditions, tissue injury, noxious heat, inflammation, or chemical stimuli like H^+^, K^+^, reactive oxygen species, and prostaglandins (Szabados et al., 2020). Following activation, these channels are permeable to ions mentioned previously resulting in membrane depolarization which can prompt the release of neuropeptides. The broad range of responses has made this channel popular to study in the cardiovascular field, though some of the findings are ambiguous and make it difficult to delineate the exact role of TRPV1 under physiologic and pathologic conditions (Horton et al., 2019).

Even though the TRPV1 receptor is expressed throughout many different organs and tissue types, it has been predominantly found and studied in sensory fibers. With respect to the cardiovascular system, TRPV1 is expressed in the vascular smooth muscle, endothelial cells, epicardial cells, and in sensory fibers that innervate the heart (Szabados et al., 2020). Expression of TRPV1 in the cardiomyocyte has been debated, but recent studies by Hong et al. (2020) identified expression using a tdTomato-GFP reporter system in mice. They did not identify fluorescence in cardiomyocytes, rather cardiac expression was only suspected in cardiac arterioles, though distinction between their presence in smooth muscle or endothelial cells in the arteries has not yet been established (Hong et al., 2020). However, studies by Gao et al. (2014) detected TRPV1 protein expression in isolated left ventricular cells from mice and H9c2 cells (Gao et al., 2014).

Concerning cardiac development, TRPV1 has been found to be an important Ca^2+^ handling protein involved in cardiomyocyte differentiation. Qi et al. examined embryonic stem cells throughout their differentiation process and TRPV1 was noted to be expressed in non-differentiated cells and had increased expression in embryonic cardiomyocytes. Agonists of TRPV1 resulted in an increase in cytosolic Ca^2+^ which is an important secondary messenger involved in cardiomyocyte development. When TRPV1 was antagonized *in vitro via* various methods, there was a decrease in the size and spontaneous activity of the cells. Antagonism of TRPV1 resulted in reduced expression of cardiomyocyte marker genes (Qi et al., 2015). In a similar model, Zhao et al. confirmed the role of TRPV1 in Ca^2+^ handling and a potential interaction with the Na^+^/Ca^2+^ exchanger (Zhao et al., 2021). These studies propose that TRPV1 may play a significant role in the Ca^2+^ handling in cardiogenesis and in early stage function.

## TRPV1 and Development of Hypertrophy

Outside of cardiac development, TRPV1 has also been investigated regarding its role in modulating cardiac hypertrophy. Myocardial hypertrophy encompasses both reversible physiological changes that occur in response to exercise as well as mostly irreversible pathological and left ventricular hypertrophy (LVH) secondary to chronic pressure overload which frequently leads to HFpEF (Iemitsu et al., 2001). In a laboratory setting, physiological hypertrophy, also termed the athletic heart, is usually modeled in animals by voluntary running (such as on a wheel), forced running (on a treadmill), or forced swimming. There are multiple models of pathological hypertrophy, but most researchers favor a transverse aortic constriction (TAC) model to induce pressure overload and LVH in mice. TRPV1 has been found to be upregulated in TAC-induced pressure overload, and mice without functional TRPV1 exhibited reduced cardiac hypertrophy (Szabados et al., 2020). Horton et al. (2013) reported that administration of a potent and selective TRPV1 antagonist, BCTC (Valenzano et al., 2003), inhibited hypertrophic remodeling and pathologic cavity dilation, while maintaining cardiac function as measured *via* ejection fraction when compared to control models. They further reported reduced heart weight/body weight (HWBW) and smaller cardiomyocyte size (Horton et al., 2013). In striking contrast, Zhong et al. used TAC pressure overload in a TRPV1^−/−^ mouse model and found genetic ablation of the channel was associated with increased LVH, increased LV cavity size, and impaired cardiac function. This was confirmed with the increased weight of the TRPV1^−/−^ hearts and a larger cardiomyocyte size (Zhong et al., 2018). Hong et al. also demonstrated an upregulation of inflammatory markers in the heart with hypertrophy development in TRPV1^−/−^ mice. They reported an increased secretion of the inflammatory cytokines TNFα and interleukin-6, both of which trigger the activation of the transcription factor NF-κB, which then stimulates the hypertrophic process. Further, TNFα is increased in hypertrophic hearts, and the receptors have been found to colocalize with TRPV1. It is believed that TNFα may increase the sensitivity of TRPV1 channels, enhancing their ability to release CGRP under pathologic conditions.

TRPV1 also appears to play an important role in mediating the fibrotic response which is a critical factor involved in the structural remodeling of the heart that is associated with the development of heart failure (HF). Both Zhong and Horton reported the effects of TRPV1 inhibition on fibrosis in their respective papers. Zhong found that genetic ablation of TRPV1 channels resulted in an increased deposition of collagen in experimental hearts. Left ventricular tissue had an increased expression of factors involved in this collagen deposition including Tgfb1, Col1a1, and Col3a1. Increased presence of fibrosis in the left ventricle was also associated with an increase in ANP, a peptide secreted by cardiac tissue under conditions of stress. In contrast with these previous findings, Horton et al. identified that antagonism of TRPV1 receptors with BCTC resulted in decreased Co3a1, a marker of collagen deposition (Horton et al., 2013).

Further studies using dietary capsaicin as a TRPV1 agonist were consistent with the findings reported by Zhong. Mice were given capsaicin enriched chow following an abdominal aortic constriction (AAC) which resulted in a decreased development of LVH and decreased expression of various hypertrophic factors, such as transforming growth factor β, phosphorylation of Smad2/3, connective tissue growth factor, and matrix metalloproteinases 2, 9, and 13. The authors identified that the activation by capsaicin reduced proliferation of fibroblasts in the heart mediated by angiotensin II. These results were not seen in TRPV1^−/−^ mice (Wang et al., 2014). Ultimately, it appears that TRPV1, likely through systemic effects, is associated with reduced fibrosis and development of hypertrophy. These findings have significant implications for the treatment, prevention, and management of heart failure as they are closely implicated in its pathophysiology.

## TRPV1 and Cardiomyopathies

TRPV1 appears to have conflicting roles regarding the development of cardiomyopathies. This duality is characterized by conflicting findings of TRPV1’s involvement in the development of cardiac hypertrophy and fibrosis. In support of a beneficial role of TRPV1, Gao et al. investigated the effects of dietary capsaicin on high salt diet in WT and TRPV1^−/−^ mice. It was observed that capsaicin improved cardiac function, reduced the development of cardiac hypertrophy and fibrosis, upregulated Peroxisome proliferation-activated receptors *δ* (PPAR-*δ*) and UCP2, and decreased inducible nitric oxide synthase (iNOS) expression in the WT mice (Gao et al., 2014). Furthermore, high salt and capsaicin fed WT mice exhibited reduced left ventricular end diastolic and systolic diameter, fractional shortening, and left ventricular posterior wall thickness compared to TRPV1^−/−^ mice that received capsaicin and controls that did not receive capsaicin. The hearts of these mice also exhibited a decreased HWBW ratio when compared to the control groups. PPAR-*δ* were found to be upregulated in the cardiomyocytes of capsaicin fed mice. PPAR-*δ* has been associated with many beneficial roles in cardiac development and function as it appears to prevent hypertrophy, protect against myocardial injury, atherosclerosis, and mitigates fibrosis by preventing fibroblast proliferation. PPAR-*δ* activation is associated with the induction of UCP2, a transport protein found in the mitochondrial membrane. Its role has yet to be clearly elucidated, but it is proposed that this protein may be associated with attenuating deleterious effects from reactive oxygen species (ROS) produced by the mitochondria (Bugger et al., 2011). Additionally, this study identified that dietary capsaicin reduced the expression of iNOS. This protein is not found in healthy heart tissue as it is associated with tissue damage (Zheng et al., 2004). iNOS expression is associated with the increased hypertrophy and fibrosis of the heart and may cause damage due to increased production of ROS secondary to the production of increased NO. Supporting these previous findings, a study by Lang et al. also used a high salt diet method and found that mice that were administered capsaicin had a reduced HWBW and exhibited less hypertrophy compared to TRPV1^−/−^ mice (Lang et al., 2015). This study further identified that the mitochondrial protein sirtuin 3 was upregulated in capsaicin fed mice. Sirtuin 3 expression, like PPAR-*δ* and UCP2, is associated with decreased ROS generation. These findings suggest that in addition to alterations of pro-fibrotic and hypertrophic factors discussed earlier, TRPV1 may be associated with upregulation of various proteins involved in attenuating oxidative stress within the heart.

In regards to potentially deleterious effects of TRPV1, various studies have associated its activation with increased hypertrophy and fibrosis contributing to the development of heart failure. Buckley and Stokes (2011) used a TAC model to induce pressure overload hypertrophy and found that mice with functional TRPV1 channels had greater HW/BW, increased end diastolic left ventricular internal diameter, reduced left ventricular ejection fraction, and bigger cardiomyocytes compared to TRPV1^−/−^ mice (Buckley and Stokes, 2011). Mice with functional TRPV1 were shown to have increased TGF-beta, a pro-fibrotic factor, caspase-3, a marker of cellular apoptosis, and atrial natriuretic peptide (Buckley and Stokes, 2011). Thilo et al. supported these findings as it was demonstrated in their study that TRPV1 expression correlated with the ventricle to body weight ratio. In addition, this study compared markers of hypertrophy in WT mice, TRPV1^−/−^ mice, and mutants that overexpressed protein phosphatase 2A (PP2Ac alpha), a protein associated with cardiac dysfunction. These mutant PP2Ac alpha mice had increased expression of TRPV1 and were associated with increased ventricle to body weight ratios and other markers of heart failure (Thilo et al., 2010).

While the function of TRPV1 in myocardium has yet to be clearly defined, many studies have investigated the expression of TRPV1 in sensory fibers and its association with the development of HF. TRPV1 has significant expression in sensory fibers and is also found on sensory afferents within the heart. In the sensory fibers of the heart, TRPV1 is involved in the cardiac sympathetic afferent reflex, which helps regulate the sympathetic tone of myocardium. Administration of certain TRPV1 agonists such capsaicin or resiniferatoxin (RTX) at high amounts can blunt capsaicin-sensitive fibers and diminish this sympathetic afferent reflex (Sanz-Salvador et al., 2012; Hong et al., 2020). Studies have shown that desensitization of these sensory fibers *via* capsaicin can diminish relaxation of the heart and even exaggerate cardiac dysfunction after induction of HF with the cardiotoxic drug Adriamycin (Szabados et al., 2020). Contrasting these results, more recent studies by Wang et al. have shown protective effects by ablating TRPV1 channels in sensory fibers. Rat models used in this study were subjected to TAC, with RTX administered locally at T1 and T4 spinal levels to ablate cardiac sensory nerves 5days before surgery. RTX is a potent TRPV1 agonist that can also blunt sensory afferents at high concentrations. The results from this study showed that RTX administration was associated with improved heart failure measures. RTX treatment decreased left ventricular end diastolic pressure and helped prevent cardiac hypertrophy, fibrosis, and apoptosis. Ejection fraction was preserved in these experimental rats (Wang et al., 2019).

In summary, TRPV1 has been extensively studied in the cardiovascular system, though its major effects appear to be through mediation of systemic and locally fibrotic effects and less so through TRPV1 channels in cardiomyocytes. These studies have largely remained in the cell and animal levels but appear to have significant clinical applications in modulating hypertrophic signaling and the development of HF.

## Transient Receptor Potential Vanilloid 2

The transient receptor potential vanilloid 2 (TRPV2) channel was initially described in Caterina et al. (1999); Kanzaki et al. (1999) and in cardiac myocytes in 2003 where it was initially referred to as growth factor-regulated channel (Iwata et al., 2003). Similar to other TRPV channels, TRPV2 consists of six transmembrane-spanning helices but is differentiated from the group by residues in its C-terminal tail (Cohen and Moiseenkova-Bell, 2014). Comparable to TRPV1, it has constrictions at both the upper and lower gates, though TRPV2 has wider constrictions in the absence of agonism in comparison with TRPV1 (Huynh et al., 2016).

TRPV2 is widely expressed in the human body with high levels of expression found in the cardiopulmonary and nervous systems as well as lymph nodes, spleen, and placenta (Kojima and Nagasawa, 2014). Specifically, it has been described in vascular smooth muscle, cardiomyocytes, and endothelial cells (Kojima and Nagasawa, 2014). Their role in smooth muscle and endothelial cells has not been fully clarified. In contrast, in cardiac cells, it has been found to be the most prominent channel in the TRPV family and is mostly localized to the sarcoplasmic reticulum and the intercalated disks where it plays an important role in modulating mechanoelectric coupling (Iwata et al., 2003).

## TRPV2 Regulation

TRPV2 has been shown in various animal models and indirectly in a handful of human studies to be an effective mechanosensitive channel that responds rapidly to stretch stimuli (Sukharev and Sachs, 2012). It transduces mechanical stretch into electrical and chemical intracellular signals and plays key roles during the maturation and development processes of various cell types (Inoue et al., 2009; Shibasaki et al., 2010) including in cardiac tissue. Using an isolated myocyte model, Iwata et al. (2003) reported that TRPV2 was upregulated and translocated to the sarcolemma (Iwata et al., 2003). Using a TAC animal model, Morine et al. (2016) and Koch et al. (2017) separately demonstrated a similar increase and translocation of the TRPV2 channel in murine cardiomyocytes derived from the left ventricle. These effects were not found to occur in response to angiotensin or beta-adrenergic stimulation (Koch et al., 2017), thus strongly supporting its role in modulating hypertrophic signaling through mechanosensitive, as opposed through intracellular signaling, pathways.

These findings were subsequently supported in clinical studies. Initially, Robbins et al. found that in patients with HF, those with larger left ventricular cavities (and likely higher myocardial stretch) exhibited higher responses to oral administration of a TRPV2 agonist (probenecid; Robbins et al., 2018). Furthermore, subjects with congenital single-ventricle physiology (where the heart is subjected to increased pressure) exhibit an upregulation of TRPV2 in comparison with normal subjects (Rubinstein et al., 2020).

TRPV2 has also been found to be upregulated in various models of muscular dystrophy (MD), a multifaceted disease that is characterized by development of progressive muscle weakness and is commonly associated with development of dilated cardiomyopathy (DCM). Various animal models have been reported to simulate MD, such as a mouse model of dystrophin-deficient Duchenne muscular dystrophy where TRPV2 overexpression was not just reported in the sarcolemma, but also in the T-tubules (Iwata and Matsumura, 2019). This finding has been supported by several publications using a hamster model of dystrophy (Iwata et al., 2018).

## TRPV2 as a Functional Protein

The increased expression of the channel in response to these genetic and external stimuli has led the field down two seemingly disparate pathways that seek to exploit its Ca^2+^ handling effects. As it pertains to the animal models of MD (as well as a few published human studies), it appears that the overexpression in cardiac tissues leads to cardiomyopathy secondary to chronically elevated intracellular Ca^2+^ levels. Regarding animal (as well as clinical) studies in cardiomyopathy and HF, this increased expression has been used to modulate Ca^2+^ handling and improve cardiac function. Thus, there is an apparent “goldilocks” effect, whereas under certain conditions an increase in expression and/or function of TRPV2 may be beneficial, but excessive TRPV2 activity may lead to Ca^2+^-dependent dysfunction and adverse cardiac remodeling.

The increased expression of TRPV2 in genetic models has led to development of various therapies that seek to *decrease* the Ca^2+^ overload frequently observed in distressed cardiomyocytes of animal models and humans with MD. These include adenoviruses, target antibodies, and previously used drugs for other diseases (Nagasawa and Kojima, 2015). These therapies have been successfully employed in animal models and in a limited number of patients (Iwata et al., 2020).

In contrast, there have been many other translational and clinical studies that seek to *stimulate* the channel to enhance Ca^2+^ cycling in the failing cardiomyocyte. These studies have mostly focused on the drug probenecid, a previously established safe compound used mostly in the treatment of gout. Probenecid has been shown to improve cardiac function in animal models of disease and humans with various forms of cardiomyopathy, focused mainly on its role in mediating Ca^2+^ handling (Koch et al., 2012, 2013; Robbins et al., 2013, 2018; Rubinstein et al., 2014; Onusko et al., 2020). However, at least one publication cites apparent anti-inflammatory effects through downregulation of the gap junction protein pannexin 1 (Good et al., 2021). More recently others have proposed cannabinoid derivatives as alternative TRPV2 channel agonists either directly through stimulation of the TRPV2 channel using compounds, such as cannabidiol or potentially indirectly *via* downstream upregulation of circulating endocannabinoids (Muller et al., 2019). These compounds are potentially fruitful avenues of clinical investigation but none have been tested directly for the cardiovascular effects on the failing myocardium (Iwata et al., 2020).

## TRPV2 as a Structural Protein

Independent of its Ca^2+^ handling effects, TRPV2 appears to play important structural roles under physiologic as well as pathologic conditions. This important observation was first suspected when the Caterina laboratory was unable to create whole gene knockout mouse models secondary to very high perinatal mortality (Park et al., 2011). This finding was confirmed many years later when a separate group developed an inducible myocyte-specific complete channel TRPV2-deficient mouse model. This model developed a severe reduction in cardiac function associated with disorganization of intercalated disks and impaired translocation of gap junction protein connexin 43 that led to cardiac dilation and associated death within 9days of induction (Katanosaka et al., 2014). This finding was in contrast to a related study seeking to understand the age related effects of TRPV2 channels in cardiac function. In order to further clarify these seemingly incompatible observations, functional-TRPV2^−/−^ (F-KO) knockout mice were developed that lack the channel pore region but maintain TRPV2 structural domains. With aging, the F-KO animals exhibited similar cardiac structure and function as WT counterparts, with an unexpected reduction in extent of myocyte hypertrophy and fibrosis (Jones et al., 2017).

Myocardial fibrosis is a critical feature of the cardiac response to pathologic stimuli including pressure overload, ischemia, and inflammation. The role that TRP channels in general, and TRPV2 specifically plays in mediating the myocyte-fibroblast interaction has been broached particularly as it pertains to the role that it plays in modulating Ca^2+^ signaling. TRPV2 has been found in cardiac fibroblasts and is capable of regulating dermal fibroblast differentiation, though its direct effects in cardiac fibroblasts as well as indirectly through myocyte-fibroblast interactions have not been studied in detail (Feng et al., 2019). Interestingly, in response to ischemic injury, TRPV2 is not only upregulated in peri-infarct cardiomyocytes (Entin-Meer et al., 2014), but also appears to play a role in modulating macrophage function under physiologic conditions and in response to ischemia under pathologic conditions (Entin-Meer et al., 2017). Thus, TRPV2-dependent blunting of the inflammatory response at both the peri-infarct level as well as systemically may be an appropriate target for regulating the fibrotic and myocardial response to injury.

In summary, there is increasing data demonstrating a dual role for TRPV2 in maintaining cardiac structure and regulating function. These findings together with potential TRPV2 effects in mediating the inflammatory response directly as well as through circulating macrophages will likely lead to novel therapeutic options in various cardiovascular diseases.

## Transient Receptor Potential Vanilloid 3

Transient receptor potential vanilloid 3 (TRPV3) was initially identified by Smith et al. (2002) who had reasoned that other TRPV channels may be located near TRPV1 in the genome. In fact, the distance between the two genes is only 7.5kb. TRPV3 is structurally homologous to the other TRPVs, containing six transmembrane regions, with cytoplasmic amino- and carboxy-termini, ankyrin domains, phosphorylation sites, and a non-selective cation permeable pore loop. TRPV3 is insensitive to capsaicin but can be heat activated at lower temperatures than TRPV1 (31–39°C compared to 42°C; Pedersen et al., 2005; Huang and Chung, 2013). TRPV1 and TRPV3 are co-expressed in dorsal root ganglion neurons, lending strength to the argument of heteromeric vanilloid receptor channels. The TRPV3 pore dilates upon stimulation to allow the passage of large cations, is modestly permeable to Ca^2+^, and has been found to be voltage-dependent (Nilius et al., 2014). TRPV3 is highly expressed in skin/keratinocytes and can also be found in brain, tongue, testis, cornea, distal colon, larynx, and inner ear. In 2018, TRPV3 expression was confirmed in the heart (Zhang et al., 2018) and recent reports suggest potential roles for TRPV3 in cardiac hypertrophy (Zhang et al., 2018; Qi et al., 2019) and fibrosis (Liu et al., 2018).

## TRPV3 in Hypertrophy and Fibrosis

As described in detail above, the hypertrophic response can be secondary to both physiologic as well as pathologic stimuli. Zhang et al. sought to determine the cardiac expression of TRPV3 in rat models of physiological and pathological hypertrophy. Their physiological hypertrophy model consisted of 90min per day forced swimming, 6days a week for 4weeks, after conditioning. The pathological hypertrophy was induced *via* a surgical AAC.

As expected, AAC produced typical pathological hypertrophy as confirmed by a decrease in left ventricular systolic pressure, an increase in left ventricular end diastolic pressure, increased HWBW and heart weight/tibia length ratios, and an increased protein expression of BNP and β-MHC. Interestingly, protein expression of TRPV3 was found to be increased in AAC hearts and IHC staining of heart tissue localized TRPV3 to the cell membrane. However, while swimming caused an increase in BWTL, verifying physiological hypertrophy, there was no increase in BNP, β-MHC, or in TRPV3 protein expression. This novel finding indicated the TRPV3 might play a role in pathological, but not physiological myocardial hypertrophy.

Zhang et al. (2018) also utilized an *in vitro* model of hypertrophy, created by perfusing neonatal rat ventricular myocytes (NRVMs) with AngII for 48h. These AngII-induced cardiomyocytes were characterized by an increase in intracellular Ca^2+^, increased phosphorylation of CaMKII, increased expression of calcineurin and translocation of NFATc3, and increased expression of BNP and TRPV3. Addition of a TRPV3 agonist, carvacrol, to the *in vitro* model resulted in cells with an increased surface area, and even higher levels of BNP, β-MHC, and TRPV3 than the model alone. Combining the *in vitro* model with carvacrol and a non-selective TRP antagonist, ruthenium red, returned the cell size, and BNP, β-MHC, and TRPV3 expression levels to that of the model alone. Target-specific knockdown of TRPV3, using siRNA, also returned the levels to that of the model and decreased the expression of phosphorylated CaMKII and the protein expression levels of calcineurin and NFATc3. Therefore, they concluded that activation of TRPV3 leads to an increase in intracellular Ca^2+^ concentration, which in turn activates calcineurin and NFATc3 to induce cardiac hypertrophy.

As part of the same laboratory, Liu et al. (2018) used a similar methodology to investigate the role of TRPV3 in cardiac fibrosis. Like Zhang et al.’s study, Liu et al. used AAC in rats and reported an increase in TRPV3. AAC rats exhibited dilation, decreased systolic function, and increased fibrosis. Addition of carvacrol further exacerbated these parameters, while addition of carvacrol and ruthenium red exhibited values similar to AAC alone. Isolation of neonatal rat ventricular fibroblast and subsequent treatment with AngII produced an *in vitro* model of cardiac fibrosis. Inclusion of carvacrol to the model increased the protein expression of TRPV3, collagen I (Col I), collagen III (Col III), TGF-β1 (transforming growth factor beta 1), Cyclin E, and CDK2 (cyclin-dependent kinase 2). Again, combining the model with carvacrol and ruthenium red was able to prevent these effects. Finally, treatment of the model fibroblasts with siRNA-TRPV3 inhibited AngII-induced increased expression of Col I and Col III, as well as Cyclin E and CDK2. Their conclusion was that TRPV3 activation promotes cyclin E and CDK2 complex expression which results in cardiac fibroblast proliferation.

The third study by Sun’s laboratory (Qi et al., 2019) sought to determine the role of miR-103 in cardiac hypertrophy, especially as it pertains to the activation of TRPV3. It has been shown that miR-103 is decreased in patients with heart failure (Ellis et al., 2013); however, the role of miR-103 in cardiac hypertrophy has not been investigated. For this study, Qi et al. utilized the AAC rat *in vivo* model, as well as the *in vitro* AngII-infused NRVM model mentioned previously. Qi et al. reasoned that the role of the TRPV3 channel in cardiac hypertrophy might involve regulation of the autophagy pathway. This idea was supported by recent publications in which aortic banding activated autophagy (Li et al., 2017; Zhao et al., 2018), decreased excessive autophagy reduced IR injury (Huang et al., 2015), and excessive autophagy caused cardiac hypertrophy (Wang and Ren, 2019). Indeed, the *in vitro* model of cardiac hypertrophy had increased levels of Beclin 2 and LC3-II and decreased levels of p62 indicating increased autophagy, which was reversed with siRNA-TRPV3. Furthermore, treatment with miR-103 decreased expression levels of TRPV3 which lead to a decreased [Ca^2+^], thereby reducing autophagy.

The therapeutic possibilities of TRPV3 agonists and antagonists have heavily focused on nociception, mainly due to the homology between TRPV1 and TRPV3 (40%), and the high expression of TRPV3 in skin keratinocytes (Huang and Chung, 2013). However, with the discovery of TRPV3 in the heart, it is expected that more studies will emerge to determine the role of TRPV3 in the cardiovascular system. One very recent study (Wang et al., 2021) found that TRPV3 is upregulated after myocardial infarction in rats and that miR-369, a microRNA regulating cardiac fibrosis, is downregulated. Further investigation using neonatal rat cardiomyocytes determined that miR-369 specifically targets TRPV3 and inhibition of TRPV3 with siRNA reduced hypoxia induced inflammation and apoptosis.

In summary, even though TRPV3 has been less studied in comparison with other TRPV channels in the heart, the evidence appears to favor the channel behaving similarly to the other TRPV channels as it pertains to its effects on regulating Ca^2+^ handling and pathologic hypertrophic signaling (as with TRPV2) as well as regulating apoptotic signaling and fibrosis (as with TRPV1). Future studies with cardiac-specific knockouts will likely shed further light on the multiple roles TRPV3 plays in regulating cardiac physiologic and pathologic responses.

## TRPV4: Discovery and Initial Characterization

Transient receptor potential vanilloid 4 (TRPV4, crystal structure Shigematsu et al., 2010; Deng et al., 2018) was initially described in four independent laboratories as the vanilloid receptor-related osmotically activated channel (Liedtke et al., 2000), osmosensitive transient receptor potential canonical 4 (OTRPC4; Strotmann et al., 2000), transient receptor potential 12 (TRP12; Wissenbach et al., 2000), and vanilloid receptor-like 2 (VRL-2; Delany et al., 2001). These disparate names were ultimately recognized as redundant of the same protein and the nomenclature reconciled to TRPV4 (Montell et al., 2002). Of these reports, cardiac TRPV4 mRNA expression was only shown by Wissenbach et al. and Strotmann et al. Subsequent work provided broad evidence of cardiac TRPV4 protein expression (Kunert-Keil et al., 2006; Shenton and Pyner, 2014; Chaigne et al., 2021) and unique localization (Zhao et al., 2012), particularly during pathologic states (Jones et al., 2019; Jia et al., 2021).

TRPV4 is an osmotically sensitive, volume regulating, non-specific [6:1 Ca^2+^/Na^+^ permeability ratio, high-conductance (50–100pS, Strotmann et al., 2003)] cation channel – unusual, as osmotically activated volume regulating channels were classically thought to be exclusively anionic (Nilius et al., 2001). Similar to other TRPV channels, TRPV4 also consists of six transmembrane-spanning α-helices with a pore loop between the fifth and sixth subunits, allowing ion permeation (Deng et al., 2018). TRPV4 normally assembles as a homotetramer (Shigematsu et al., 2010; Stewart et al., 2010); however, heterotetrameric assembly has been extensively reported (Stewart et al., 2010; Ma et al., 2011; Du et al., 2014; Greenberg et al., 2017). Despite initial descriptions as exclusively osmotically sensitive, TRPV4 was subsequently found to be promiscuously gated *via* heat, osmolarity, ligand activation, pH, and stretch, among other stimuli (Güler et al., 2002; Watanabe et al., 2002; Nilius et al., 2003; Smith et al., 2006; Plant and Strotmann, 2007; Vriens et al., 2009). Given TRPV4 selectivity for Ca^2+^ and high ionic flux, recent reports have shown functional roles for cardiac TRPV4, including in the myocardium and cardiac fibroblasts.

## TRPV4 in Myocardial Tissue

Initial experiments postulating functional roles for myocardial TRPV4 were conducted by Li et al. using papillary muscle exposed to hyper- and hypo-osmotic conditions and measuring force output (Li et al., 2008). 4α-phorbol 12, 13-didecanoate (4αPDD; Watanabe et al., 2002), a phorbol ester derivative, was used for TRPV4 agonism and ruthenium red for potential TRPV4 antagonism to show TRPV4-dependent contribution to osmotic modulation of cardiac contractility. While 4αPDD selectivity for TRPV4 was first posited by Watanabe et al. (2002), subsequent work by Alexander et al. (2013) showed 4αPDD excitation independent of TRPV4 (Watanabe et al., 2002; Alexander et al., 2013). Furthermore, ruthenium red has been extensively shown to be non-selective (Watanabe et al., 2002; Lee et al., 2013). The investigations by Li et al., while ultimately inconclusive owing to problems of pharmacological specificity, set the stage for further work elucidating the functional role of myocardial TRPV4 (Li et al., 2008).

Qi et al. furthered investigation of TRPV4-dependent myocardial intracellular Ca^2+^ influx using uniaxial cell stretch in human embryonic stem cell cardiomyocytes (Qi et al., 2015). While non-specific TRPV agonist 4αPDD increased fura-2 assayed intracellular Ca^2+^, TRPV4-specific antagonists RN1734 and HC067047 partially attenuated the increase and non-specific channel inhibitor ruthenium red nearly abolished the increase. Lentivirus vectors with dominant-negative TRPV4 similarly attenuated 4αPDD-mediated increases in intracellular Ca^2+^, further demonstrating TRPV4 specificity. This work was confirmed by Lu et al. who also observed increased TRPV4-dependent intracellular Ca^2+^ in human induced pluripotent stem cell-derived (hiPSC) cardiomyocytes undergoing uniaxial stretch (Lu et al., 2017). However, in addition to control hiPSC cardiomyocytes, they subjected hiPSC cardiomyocytes from DCM patients to the same stretch protocol and found stretch and TRPV4-dependent increases in intracellular Ca^2+^ over that seen in control hiPSC cardiomyocytes, revealing a possible role for TRPV4 in mediating myocardial pathology. Indeed, under excessive stretch conditions (stretching cells 30% beyond resting length), hiPSC cardiomyocytes from DCM patients displayed prolonged Ca^2+^ transients and oscillations in Ca^2+^, which were attenuated by TRPV4 antagonism. While these two groups showed a role for stretch-induced TRPV4 activation and subsequently increased intracellular Ca^2+^ in both physiologic and pathophysiologic states, work showing a functional role for TRPV4 in primary tissue was still lacking.

The first study to demonstrate a critical role for TRPV4 in mediating myocardial ischemia/reperfusion (I/R) injury was performed in mice with left anterior descending (LAD) coronary artery ligation, in the presence and absence of TRPV4-specific inhibitor HC067047 (Dong et al., 2017). Both TRPV4 mRNA and protein expression were shown to be elevated in a time-dependent manner following I/R injury, and TRPV4 antagonism decreased infarct size and increased ejection fraction 24h following I/R. Further, these improvements were shown with TRPV4 antagonism either before or within a four-hour timeframe following I/R. Perhaps most importantly, TRPV4 antagonism reduced I/R induced cardiomyocyte apoptosis as assayed by TUNEL staining. These findings were also recapitulated in global TRPV4^−/−^ mice. Similar results were obtained from the group’s subsequent paper demonstrating more detailed mechanisms of TRPV4 involvement in I/R injury using H9C2 and neonatal rat ventricular myocytes subjected to hypoxia in culture (Wu et al., 2017). As before, TRPV4 antagonism improved outcomes following hypoxia/reperfusion injury and the group additionally demonstrated the effect of TRPV4 agonism on exacerbating hypoxia/reperfusion and ischemia/reperfusion injury in cells and whole hearts, respectively. The mechanism for alleviation of TRPV4-dependent I/R and H/R injury in these models was later shown (by the same group) to involve improved antioxidation and decreased reactive oxygen species generation (Wu et al., 2019).

While the role for TRPV4 in cardiac physiology was emerging *in vivo* and in cultured cell systems, the mechanistic role of TRPV4 in directly modulating cardiomyocyte Ca^2+^ fluxes in adult cardiomyocytes remained unclear. To elucidate the contribution of TRPV4 to cardiomyocyte Ca^2+^homeostasis, Jones et al. enzymatically isolated primary left ventricular cardiomyocytes from hearts of young (3–6month) to aged (24–27month) mice. TRPV4 protein expression was increased in cardiomyocytes of aged mice, and following hypo-osmotic stress cardiomyocytes of aged, but not young, responded with an increase in Ca^2+^ transients, increased Ca^2+^ spark frequency, and elevated sarcoplasmic reticulum Ca^2+^ content (Jones et al., 2019). Hypo-osmotic stress-induced changes in intracellular Ca^2+^ homeostasis were abolished by TRPV4 antagonism *via* HC067047. Similar effects on Ca^2+^ homeostasis were induced in young mice with transgenic cardiac-specific TRPV4 Overexpression. Using Langendorff perfused hearts subjected to global I/R, aged (but not young) heart experienced a phase of hypercontractility early in reperfusion. The hypercontractile phase was prevented by TRPV4 inhibition with HC067047 and absent in global TRPV4(^−/−^). TRPV4 antagonism with HC067047 also reduced cardiac damage of aged hearts following I/R.

Changing TRPV4-stimulus modality, Veteto et al. explored the contribution of TRPV4 to myocardial stretch-induced cardiomyocyte Ca^2+^ entry, left ventricular pressure development, and cell survival in the context of aging using both isolated primary left ventricular cardiomyocytes and isolated working heart preparations (Veteto et al., 2020). Isolated working hearts of aged mice challenged with preload elevations exhibited both initial Frank-Starling and secondary increases in developed pressure that were attenuated by TRPV4 antagonism using HC067047. This secondary hypercontractility was temporally distinct from both previously described stretch-induced inotropy mechanisms, the Frank-Starling Law of the Heart, and the ANREP/slow-force response (von Anrep, 1912; Starling and Visscher, 1927; Frank, 1959). With sustained preload challenge, however, hearts of aged mice exhibited a rapid loss of systolic and diastolic function suggestive of stretch-induced damage. Consistent with this notion, isolated cardiomyocytes of aged hearts subjected to 10–15% stretch also showed initial increased maximal systolic and diastolic intracellular Ca^2+^ that preceded terminal contracture and cell damage. Both the preload-induced loss in contractile function in isolated hearts and the stretch-induced terminal contracture in isolated cells were reduced by TRPV4 antagonism with HC067047.

Though significant work from several labs showed increased TRPV4-dependent intracellular Ca^2+^ flux including increased diastolic Ca^2+^, prolonged Ca^2+^ transients, and decay times, Liao et al. were the first group to explicitly show that TRPV4 mediates atrial cardiomyocyte arrhythmogenesis and atrial fibrillation in sterile pericarditis rats (Liao et al., 2020). Pacing induced atrial fibrillation (AF) 3days post-pericardiotomy was ameliorated in both frequency and duration by treatment with the TRPV4-specific inhibitor GSK2193874. Likewise, AF incidence in isolated hearts was decreased. Action potential duration (APD) increased following pericardiotomy was also shortened using TRPV4 antagonism. These results suggest TRPV4 plays an arrhythmogenic role in AF *via* induction of adverse atrial remodeling and/or changes in atrial cardiomyocyte Ca^2+^ flux.

Chaigne et al. followed up with an investigation into the role of TRPV4 in modulating cardiac electrophysiology in mice (Chaigne et al., 2021). After demonstrating TRPV4 colocalization with the L-type Ca^2+^ channel (Ca_v_1.2) in isolated ventricular cardiomyocytes, subsequent *in vivo* ECGs revealed TRPV4-dependent QT prolongation. This led to patch clamp investigation of isolated ventricular cardiomyocytes from WT (TRPV4 expressing) and TRPV4 knockout mice (TRPV4^−/−^) wherein action potential duration at 90% repolarization (APD_90_) was significantly shorter in TRPV4^−/−^ mice. TRPV4 agonism with GSK1016790A further increased APD_90_ which was attenuated by TRPV4 antagonism with GSK2193874 and absent in TRPV4^−/−^ isolated ventricular cardiomyocytes. Ca^2+^ transient amplitude was also attenuated in TRPV4^−/−^ isolated ventricular cardiomyocytes, further confirming TRPV4 involvement in modulating ventricular cardiomyocyte Ca^2+^ flux. The findings of prolonged QT and APD_90_ *in vivo* and *in vitro* suggest a possible role for TRPV4 in mediating ventricular arrhythmia similar to the previous study of TRPV4-associated arrhythmogenesis in AF by Liao et al. At around the same time, Peana et al. used sharp electrode electrophysiology in isolated perfused hearts of aged mice (+/−TRPV4 inhibition) to examine TRPV4-dependent effects on cardiomyocyte membrane potential following LAD ligation I/R (Peana et al., 2021). Following I/R, TRPV4 led to membrane potential depolarization, slower action potential kinetics, and increased incidence of ventricular arrhythmia. Furthermore, high-resolution confocal imaging was used to image sub-epicardial cardiomyocytes in isolated hearts of aged mice with cardiac-specific expression of the GCaMP6f Ca^2+^ indicator. In these studies, global I/R induced pro-arrhythmic Ca^2+^ overload that was prevented using TRPV4 inhibition with HC067047. This study showed the role of TRPV4 in age-dependent arrhythmia following I/R, suggesting a possible therapeutic target for TRPV4 following myocardial infarction in aging.

## TRPV4 and Cardiac Fibroblast

Similar to Li et al., Hatano et al. first characterized TRPV4-dependent intracellular Ca^2+^ flux modulation in cardiac fibroblasts using ruthenium red, 4αPDD, and siRNA TRPV4 knockdown (Hatano et al., 2009). Cardiac fibroblasts play integral roles in extracellular matrix (ECM) deposition and hormone release as well as in structural remodeling following myocardial injury (Brilla and Maisch, 1994; Banerjee et al., 2006). While compensatory structural remodeling is critical to prolonged cardiac function, excessive cardiac fibroblast proliferation and activation can lead to elevated myocardial stiffness and predispose hearts to diastolic dysfunction (Biernacka and Frangogiannis, 2011; Frangogiannis, 2021). This activation and proliferative signaling are in part due to increased intracellular Ca^2+^ flux in cardiac fibroblasts. In their experiments, Hatano et al. isolated cardiac fibroblasts and used whole-cell patch clamp and fura-2 fluorescence experiments to examine the effects of TRPV4 agonism/antagonism on Ca^2+^ current and intracellular Ca^2+^ concentration. These experiments showed 4αPDD increased both intracellular Ca^2+^ and current density and that ruthenium red attenuated these elevations. Crucially, Hatano et al. further employed TRPV4 knockdown using siRNA to show that 4αPDD-dependent increases in intracellular Ca^2+^ concentration and current density were at least partially TRPV4-dependent (Hatano et al., 2009). This demonstrated TRPV4-dependent increases in intracellular Ca^2+^ associate with cardiac fibroblast differentiation.

Building on the work of Hatano et al., Adapala et al. utilized novel pharmacologic tools to determine how TGFß1-induced TRPV4 activation and the subsequently increased intracellular Ca^2+^ flux affects cardiac fibroblast activation and differentiation into myofibroblasts, the cellular entities primarily responsible for ECM deposition, and remodeling following myocardial infarction (Biernacka and Frangogiannis, 2011; Adapala et al., 2013). While cardiac fibroblast to myofibroblast transition is a physiologic part of scar formation and cardiac remodeling, uncontrolled differentiation, proliferation, and activation of myofibroblasts can lead to pathologic fibrosis and concomitant diastolic dysfunction. Advances in TRPV4-specific agonists and antagonists prior to this work permitted more pharmacologic specificity in answering questions about TRPV4’s contribution to these processes (Vincent et al., 2009; Vriens et al., 2009; Vincent and Duncton, 2011). Isolated rat left ventricular cardiac fibroblasts were subjected to TGFß1 and both intracellular Ca^2+^ and αSMA stress fiber deposition monitored as indications of cardiac fibroblast activation and myofibroblast differentiation, respectively. In both protocols, TRPV4 antagonism or knockdown (using siRNA) reduced activation and αSMA deposition, whereas TRPV4 agonism increased activation and myofibroblast differentiation. These experiments therefore showed that TRPV4 activation directly contributes to myofibroblast differentiation and posited the possibility of aberrant TRPV4 activation leading to pathologic remodeling.

In tandem with I/R and osmotic stress experiments in cardiomyocytes, Adapala et al. investigated TRPV4-dependent adverse remodeling following myocardial infarction *via* cardiac fibroblast differentiation (Adapala et al., 2013). Eight weeks following LAD ligation, WT mice showed markedly reduced cardiac function as assayed by echocardiography that was partly attenuated in TRPV4 knockout mice. TRPV4 knockout mice demonstrated improved survival compared to their WT counterparts and picrosirius red staining revealed decreased fibrotic deposition in the TRPV4 knockout hearts following MI. Given the whole organ response to induced MI, isolated cardiac fibroblasts were then subjected to hypo-osmotic stress and monitored for intracellular Ca^2+^ flux using fluo-4. Cardiac fibroblasts from WT hearts displayed increased intracellular Ca^2+^ that was absent in cardiac fibroblasts from TRPV4 knockout hearts. This correlated with decreased TGF-ß1-induced myofibroblast differentiation in cultured cardiac fibroblasts isolated from TRPV4 knockout hearts, which was shown to be mediated by the Rho/Rho kinase signaling pathway following I/R. Therefore, Adapala et al. demonstrated the functional role of TRPV4 in mediating adverse cardiac remodeling following myocardial infarction at the *in vivo*, tissue, and cell level.

## Conclusion and Translational Outlook

In summary, the study of TRPV channels in cardiovascular has significantly grown over the past decade with significant improvements in our understanding of their function under physiologic and pathologic conditions, particularly those involving increased stress on the cardiomyocyte. This improved knowledge has expanded the field to include various cardiovascular cell types, most prominently cardiac fibroblasts, and has led to a number of potentially clinically relevant therapeutic options for patients with or at risk of heart disease.

Currently, there are at least two clinically relevant applications that are being tested in patients and a handful more that are likely to be studied in the near future. The current studies both focus on TRPV2 channels though in different directions as described above. The blockade of TRPV2 is likely to yield interesting findings in the treatment and potentially prevention of cardiomyopathy in patients with muscular dystrophy, though as with all TRPV-related studies, it will be crucial to establish the dose and timing of the modulation of the channel. The activation of TRPV2 for patients with heart failure is ongoing and has recruited a handful of patients. The data for this prospective study will likely not be available for several years as the treatment phase is 6months and many more patients will need to be recruited.

Upcoming studies related to TRPV channels will likely include the channels mostly associated with ischemia and inflammation, such as those in the sensory fibers (TRPV1 and TRPV3) that modulate cardiovascular tone and potentially the preconditioning response and those in macrophages (TRPV2) that modulate the inflammatory response during and following a myocardial infarction. Lastly, as all TRPV channels modulate Ca^2+^ influx, there is potential for their use in the treatment and prevention of arrhythmias, particularly post-ischemia as described for TRPV4.

## Author Contributions

JR and TD contributed to the original review topic. MM, SK, AV, TD, and JR contributed to writing various sections of the review. SK, TD, and JR edited the review. MM and TD contributed to the schematic design created with BioRender.com. All authors contributed to the article and approved the submitted version.

## Conflict of Interest

AV is employed by the IonOptix LLC.The remaining authors declare that the research was conducted in the absence of any commercial or financial relationships that could be construed as a potential conflict of interest.

## Publisher’s Note

All claims expressed in this article are solely those of the authors and do not necessarily represent those of their affiliated organizations, or those of the publisher, the editors and the reviewers. Any product that may be evaluated in this article, or claim that may be made by its manufacturer, is not guaranteed or endorsed by the publisher.
